# Who is at risk? Clinical features and a predictive model for 30-day mortality in hematologic patients with enterococcal bloodstream infection

**DOI:** 10.3389/fcimb.2026.1762404

**Published:** 2026-03-03

**Authors:** Nuobing Yang, Sisi Zhen, Tingting Zhang, Yuping Fan, Qingsong Lin, Yingchang Mi, Yizhou Zheng, Lugui Qiu, Fengkui Zhang, Erlie Jiang, Mingzhe Han, Zhijian Xiao, Jianxiang Wang, Sizhou Feng, Xin Chen

**Affiliations:** 1 State Key Laboratory of Experimental Hematology, National Clinical Research Center for Blood Diseases, Haihe Laboratory of Cell Ecosystem, Institute of Hematology and Blood Diseases Hospital, Chinese Academy of Medical Sciences and Peking Union Medical College, Tianjin, China; 2 Tianjin Institutes of Health Science, Tianjin, China

**Keywords:** breakthrough infections, enterococcus bloodstream infection, hematologic diseases, mucosal barrier injury laboratory-confirmed bloodstream infections, prognostic model

## Abstract

**Background:**

Enterococcal bloodstream infection (EBSI) carries high mortality in hematologic patients, yet no prognostic model tailored to this population exists.

**Methods:**

We retrospectively analyzed 192 hematologic patients (≥14 years) with EBSI admitted between 2014 and 2024. Clinical features, microbiology, treatment, and outcomes were assessed. Candidate predictors for 30-day mortality were selected by LASSO and entered into multivariable logistic regression. A simplified risk score was derived from regression coefficients and internally validated by bootstrap resampling.

**Results:**

The median patient age was 43 years, and acute leukemia was the predominant underlying disease (72.4%). *Enterococcus faecium* was the leading pathogen (71.4%), with low vancomycin resistance (1.6%). Most cases (71.9%) occurred as breakthrough infections, mainly during carbapenem therapy, and 72.9% met mucosal barrier injury laboratory-confirmed bloodstream infection criteria. The 14- and 30-day all-cause mortality rates were 13.5% and 22.4%, respectively. Independent predictors of 30-day mortality included age ≥50 years (aOR=2.29, p=0.038), severe graft-versus-host disease (aOR=6.06, p=0.003), septic shock (aOR=30.01, p<0.001). The final predictive model, incorporating these three factors along with pneumonia and high-risk hematologic disease, demonstrated optimal discrimination (AUROC 0.79, 95% CI 0.705–0.867) and calibration. A derived risk score stratified patients into low- (<2 points) and high-risk (≥2 points) groups, with markedly different 30-day mortality (11.3% *vs*. 39.0%, P<0.001).

**Conclusions:**

In hematologic patients, EBSIs commonly arise as breakthrough infections despite broad-spectrum antibiotic coverage, most often associated with mucosal barrier injury. Our parsimonious risk score enables early identification of patients at high risk of 30-day mortality to guide timely interventions.

## Introduction

Enterococcal bloodstream infection (EBSI) is common in patients with hematologic diseases, probably due to immunosuppression, extensive use of central venous catheters (CVCs) or peripherally inserted central catheters (PICCs), and the empiric administration of broad-spectrum antibiotics like cephalosporins, which are primarily targeted against gram-negative bacteria and exhibit limited activity against enterococci (Satlin et al., 2014; Misch and Andes, 2019). Enterococci constitute a component of the endogenous intestinal microbiota. In hematologic patients undergoing chemotherapy or hematopoietic stem cell transplantation (HSCT), injury to the intestinal mucosal barrier may facilitate bacterial translocation, leading to subsequent bloodstream infection.

Reported mortality rates for EBSI in this vulnerable population range from 22% to 45% (Vydra et al., 2012; Bae et al., 2019; Papanicolaou et al., 2019). Kalaycio et al. further demonstrated that early vancomycin-resistant enterococcal (VRE) bacteremia following allogeneic HSCT (allo-HSCT) is associated with a rapidly deteriorating clinical course (Avery et al., 2005). Despite these concerning outcomes, no prognostic model currently exists that is specifically designed for hematologic patients with EBSI. Most available prediction tools have primarily focused on patients with gram-negative bacteremia (Tang et al., 2018; Li et al., 2025). To address this gap, we sought to develop a prognostic model tailored specifically to hematologic patients with EBSI.

In this study, we investigated the clinical characteristics, management, and outcomes of EBSI in patients (≥14 years) with hematologic diseases. Furthermore, we developed and internally validated a simple prognostic scoring system to predict patient outcomes at the early stage of bacteremia, thereby supporting timely risk stratification and guiding individualized clinical interventions.

## Method

### Setting and patients

This retrospective study was conducted at a specialized hematology hospital in Tianjin, China, between January 2014 and December 2024. Patients were eligible if they were ≥14 years old, had at least one positive blood culture for *Enterococcus* spp., and had complete medical records with 30-day follow-up data. In cases of a second episode of EBSI occurring within 90 days of the initial episode, only the first episode was included for analysis; subsequent episodes were classified as recurrences. This study was approved by the Ethical Committee of the Institute of Hematology and Blood Diseases Hospital, Chinese Academy of Medical Sciences and Peking Union Medical College.

### Data collection

Demographic and clinical data were retrieved from the electronic medical records, including type and phase of hematologic disease; chemotherapy or immunosuppressive therapy; allogenic or autologous HSCT (allo-HSCT, auto-HSCT); presence of grade II-IV acute graft-versus-host disease (aGVHD) or moderate-to-severe chronic graft-versus-host disease (cGVHD) (Jagasia et al., 2015; Schoemans et al., 2018); Charlson comorbidity index (CCI); absolute neutrophil count (ANC); duration of neutropenia before and after bacteremia; suspected infection source; site of acquisition; occurrence of septic shock; microbiological data (species, vancomycin and ampicillin resistance); follow-up blood culture results (recommended 48–96 h after antibiotic initiation (Lopez-Cortes et al., 2013)); antibiotic regimens; and clinical outcomes.

In accordance with current guidelines (Rosselli Del Turco et al., 2021), echocardiography was recommended at our center for EBSI patients meeting any of the following criteria: recent HSCT, community-acquired EBSI, prolonged fever or recurrent fever after initial defervescence, persistent or recurrent bacteremia, a predisposing condition for endocarditis (e.g., native valve disease, prosthetic valve, or any cardiac implantable electronic devices), clinical signs of endocarditis (e.g., embolic events, conjunctival hemorrhage, Janeway lesions and immunologic phenomena), or evidence of cardiac dysfunction.

### Outcomes

The primary outcome was all-cause mortality within 30 days of the first positive blood culture. Secondary outcomes included 14-day all-cause mortality, infection-related mortality and 90-day recurrence. Death was considered infection-related if it occurred before resolution of signs or symptoms, or within 7 days of bacteremia onset, without other identifiable causes (Lopera et al., 2024). Recurrence was defined as a microbiologically confirmed *Enterococcus*-positive blood culture with the same species and resistance profile as the initial isolate, occurring within 90 days of the initial EBSI episode and after completion of antibiotic therapy (Peterson et al., 2009; Cattaneo et al., 2021).

### Definitions

The onset of EBSI was defined as the date the first positive blood culture sample was collected. Neutropenia was defined as an ANC < 0.5×10^9^/L, and severe neutropenia as ANC < 0.1×10^9^/L. Hematologic diseases were categorized into three groups: bone marrow failure syndromes (aplastic anemia [AA] and myelodysplastic syndromes [MDS]), acute leukemia (acute myeloid leukemia [AML], acute lymphoblastic leukemia [ALL] and mixed phenotype acute leukemia [MPAL]), and other hematologic diseases. Standard-risk disease was defined as acute leukemia in complete remission (CR); lymphoma or multiple myeloma in CR or partial remission (PR); MDS with <5% blasts; or untreated severe aplastic anemia (SAA). High-risk disease included newly diagnosed acute leukemia, induction failure or relapse, lymphoma or multiple myeloma with stable disease or progression, as well as MDS/SAA with transfusion dependence and no response to treatment (Wang et al., 2015; Zhang et al., 2023). Grade II-IV aGVHD or moderate-to-severe cGVHD was collectively defined as severe GVHD.

Acquisition of EBSI was classified as: (i) community-onset, defined as a positive blood culture obtained at or within 48 h of hospital admission; (ii) nosocomial, defined as a positive blood culture obtained ≥48 h after hospitalization. According to the Centers for Disease Control and Prevention (CDC) criteria, the source of bacteremia was categorized as mucosal barrier injury laboratory- confirmed bloodstream infection (MBI-LCBI), non–MBI primary EBSI, or secondary EBSI (Centers for Disease Control and Prevention, 2025). For patients with an eradicable focus, appropriate source control interventions (e.g., abscess drainage, excision, or catheter removal) were performed. Breakthrough EBSI was defined as the occurrence of EBSI in patients who were receiving systemic antimicrobial therapy for at least 48 hours prior to the collection of the index positive blood culture (Rangaraj et al., 2010). Septic shock was defined as systolic pressure <90 mmHg despite adequate fluid resuscitation or the need for vasopressor agents (Rhodes et al., 2017). Persistent bacteremia was defined as positive blood cultures for *Enterococcus* spp. persisting ≥72 h after initiating appropriate antibiotic therapy. Metastatic infection was defined as definite infective endocarditis fulfilling the modified Duke criteria (Li et al., 2000) or a secondary infection at a site distant from the primary focus. Polymicrobial bacteremia was defined as the isolation of *Enterococcus* spp. with one or more additional bacterial species from the same or another blood culture obtained within 24 h, meeting CDC criteria for bloodstream infection (Centers for Disease Control and Prevention, 2025). Appropriate antibiotic therapy was defined as administration of at least one *in vitro*-active agent against the *Enterococcus* isolate. For polymicrobial bacteremia, appropriate therapy required coverage of all identified pathogens.

### Microbiological studies

Clinical samples were processed at the hospital microbiology laboratory using an automated VITEK 2 Compact system for species identification and susceptibility testing. Antibiotic susceptibilities were defined according to current Clinical and Laboratory Standards Institute (CLSI) criteria.

### Statistical analyses

Categorical variables were compared using Chi-square test or Fisher’s exact test, as appropriate. Continuous variables were analyzed using the Mann-Whitney U test for nonparametric data. A two-tailed P value <0.05 was considered statistically significant.

Candidate predictors of 30-day mortality were identified using least absolute shrinkage and selection operator (LASSO) logistic regression. All candidate variables were restricted to those available within 72 hours after blood culture collection, as most *Enterococcus*-positive results are available within this timeframe. Predictors with non-zero coefficients at the minimum cross-validated lambda were retained. A multivariable logistic regression model including variables selected by LASSO was initially constructed. To enhance parsimony and clinical interpretability, nested models were subsequently compared. Variables whose exclusion did not adversely affect model discrimination or calibration were excluded. The final model was determined based on overall predictive performance (area under the receiver operating characteristic curve [AUROC], calibration, Brier score, decision curve analysis [DCA]) and model simplicity.

A clinical risk score was derived from the regression coefficients of the final multivariable model. The detailed calculation method is provided in the Supplementary Methods. Internal validation of both the regression model and the point-based score was performed using 2000-bootstrap resampling. The optimal cutoff value for risk stratification was determined using ROC curve analysis of the total risk score, applying the Youden index. Because the risk score was integer-based, the identified cutoff was rounded up to the nearest integer for clinical applicability. Data analyzes were performed using R software version 4.5.1.

## Results

### Patient characteristics

From 2014-2024, a total of 192 patients (≥14 years) with hematologic diseases complicated by EBSI were included. Baseline clinical characteristics are summarized in **Table 1**. The median age was 43 years (interquartile range [IQR], 31.0–55.0), and 57.3% (n=110) were male. Acute leukemia was the most common underlying hematologic disease (n=139, 72.4%), including AML (n=96), ALL (n=39), and MPAL (n=4). Bone marrow failure syndromes accounted for 38 patients (19.8%), comprising MDS (n=18) and AA (n=20). The remaining 15 patients (7.8%) had other hematologic disorders, including multiple myeloma (n=3), Evans syndrome (n=1), lymphoma (n=6), and hemophagocytic lymphohistiocytosis (n=5). Ninety patients (46.9%) had high-risk hematologic diseases. Most patients (n=178, 92.7%) had received chemotherapy or immunosuppressive therapy within 1 month prior to EBSI. Within 100 days prior to EBSI, 44 (22.9%) and 6 (3.1%) patients had undergone allo-HSCT and auto-HSCT respectively. Fifteen patients (7.8%) had severe GVHD at the time of EBSI, including 13 with grade II-IV aGVHD and 2 with severe cGVHD. The 30-day mortality rate was significantly higher in patients with severe GVHD than those without (46.7% [7/15] *vs*. 20.3% [36/177], p=0.043).

**Table 1:** Comparison of patients who survived or not at day 30 after enterococcal bloodstream infection onset.

Type of variable, characteristic	Overall (n=192)	30d non-survivors (n=43)	30d survivors (n=149)	P
Age	43.0 [31.0, 55.0]	52.0 [37.0, 58.0]	42.0 [29.5, 54.0]	0.023
≥50 (%)	77 (40.1)	25 (58.1)	52 (34.9)	0.010
Male (%)	110 (57.3)	27 (62.8)	83 (55.7)	0.408
CCI	2.0 [2.0, 2.0]	2.0 [2.0, 3.0]	2.0 [2.0, 2.0]	0.245
Type of hematologic disease (%)				0.042
Bone marrow failure syndromes	38 (19.8)	10 (32.2)	28 (18.8)	
Acute leukemia	139 (72.4)	26 (60.5)	113 (75.8)	
Other hematological diseases	15 (7.8)	7 (16.3)	8 (5.4)	
Stage of underlying diseases (%)				0.093
Standard risk	102 (53.1)	18 (41.9)	84 (56.4)	
High risk	90 (46.9)	25 (58.1)	65 (43.6)	
Allo-HSCT, past 100d (%)	44 (22.9)	8 (18.6)	36 (24.2)	0.445
Severe GVHD (%)	15 (7.8)	7 (16.3)	8 (5.4)	0.043
Auto-HSCT, past 100d (%)	6 (3.1)	0 (0.0)	6 (4.0)	0.341
Chemotherapy or immunosuppressive therapy within 1 month prior to EBSI (%)	178 (92.7)	38 (88.4)	140 (94.0)	0.364
Microbiology (%)				0.364
Enterococcus faecalis	40 (20.8)	9 (20.9)	31 (20.8)	
Enterococcus faecium	137 (71.4)	33 (76.7)	104 (69.8)	
Other enterococci	15 (7.8)	1 (2.3)	14 (9.4)	
Ampicillin resistance (%)	130 (67.7)	30 (69.8)	100 (67.1)	0.743
Vancomycin resistance (%)	3 (1.6)	1 (2.3)	2 (1.3)	0.535
Polymicrobial bacteremia (%)	34 (17.7)	12 (27.9)	22 (14.8)	0.047
Breakthrough bacteremia (%)	138 (71.9)	31 (72.1)	107 (71.8)	1.000
Days of antibiotic use before breakthrough EBSI	9.0 [5.0, 12.0]	9.0 [6.5, 13.5]	8.0 [5.0, 12.0]	0.307
Source of infection (%)				0.447
MBI-LCBI	140 (72.9)	33 (76.7)	107 (71.8)	
Non-MBI primary BSI	18 (9.4)	5 (11.6)	13 (8.7)	
Secondary BSI	34 (17.7)	5 (11.6)	29 (19.5)	
Nosocomial infection (%)	184 (95.8)	40 (93.0)	144 (96.6)	0.539
Metastatic infection (%)	9 (4.7)	1 (2.3)	8 (5.4)	0.673
Persistent bacteremia (%)	15 (7.8)	5 (11.6)	10 (6.7)	0.462
Septic shock (%)	12 (6.3)	11 (25.6)	1 (0.7)	<0.001
Pneumonia (%)	93 (48.4)	28 (65.1)	65 (43.6)	0.013
Day 1 ANC 0–500 cells/mL (%)	162 (84.4)	37 (86.0)	125 (83.9)	0.732
Day 1 ANC 0–100 cells/mL (%)	145 (75.5)	29 (67.4)	116 (77.9)	0.162
Days of neutropenia before BSI	12.0 [4.0, 19.3]	12.0 [3.0, 21.5]	11.0 [4.0, 19.0]	0.649
Days of neutropenia after BSI	7.0 [3.0, 13.0]	5.0 [3.0, 10.0]	7.0 [3.0, 14.0]	0.096
Inappropriate therapy within 24 h (%)	113 (58.9)	29 (67.4)	84 (56.4)	0.194
Inappropriate therapy within 48 h (%)	68 (35.4)	18 (41.9)	50 (33.6)	0.316
Inappropriate therapy within 72 h (%)	15 (7.8)	4 (9.3)	11 (7.4)	0.928
Duration of antibiotic therapy	12.0 [8.0, 17.0]	6.0 [3.0, 12.5]	12.0 [9.0, 18.0]	<0.001

CCI, Charlson Comorbidity Index; allo-HSCT, allogeneic hematopoietic stem cell transplantation; GVHD, graft-versus-host disease; auto-HSCT, autologous hematopoietic stem cell transplantation; BSI, bloodstream infection; MBI-LCBI, mucosal barrier injury laboratory- confirmed bloodstream infection; ANC, absolute neutrophil count.

### Microbiology and clinical manifestations

**Figure 1:**
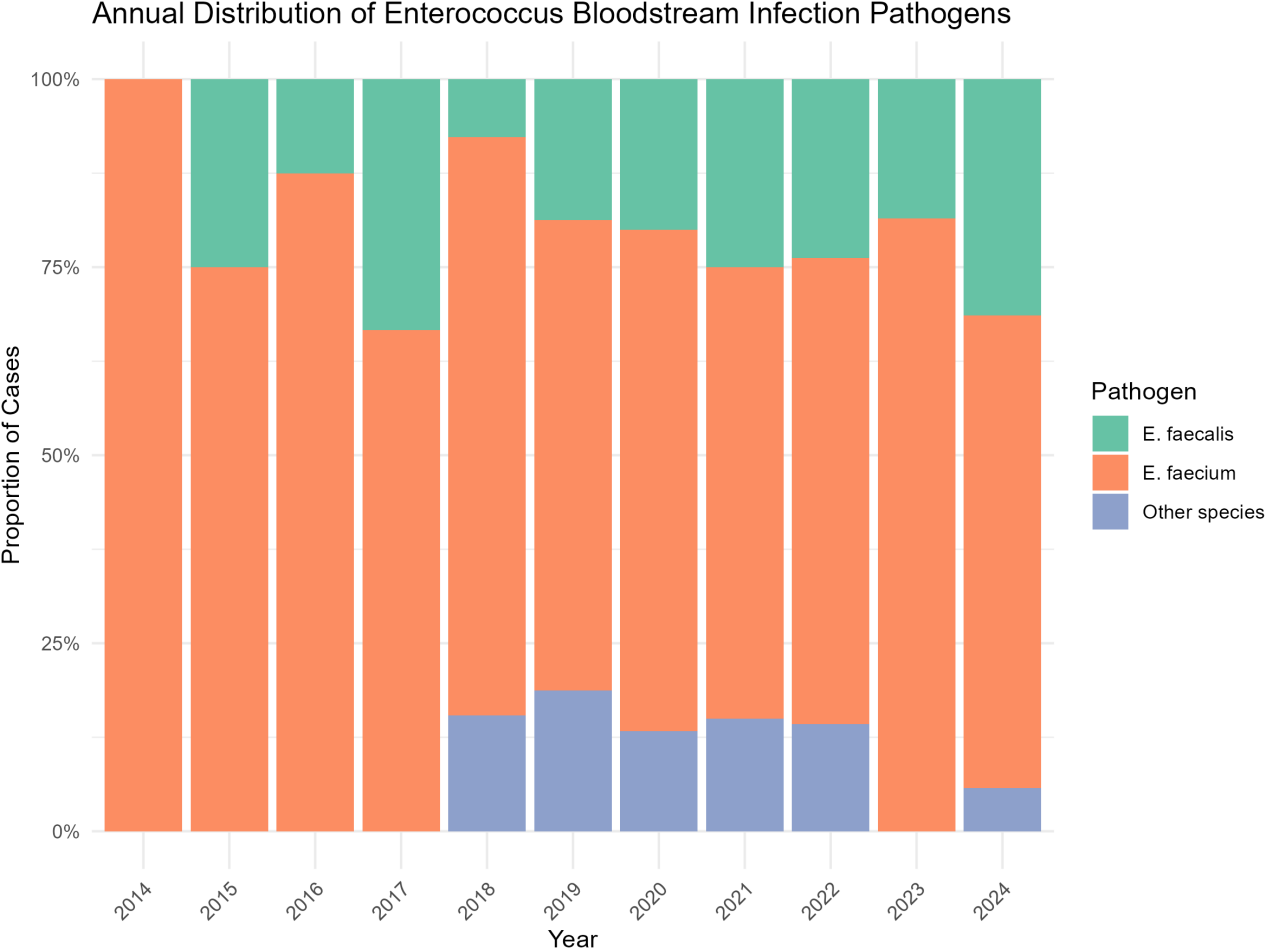
Annual distribution of enterococcus bloodstream infection pathogens.

*Enterococcus faecium* was the predominant pathogen (n=137, 71.4%), followed by *Enterococcus faecalis* (n=40, 20.8%) and other *Enterococcus* species (n=15, 7.8%). The temporal distribution of species is shown in **Figure 1**. Polymicrobial bloodstream infection was identified in 34 patients (17.7%). The detaileddistribution of the accompanying pathogens is summarized in **Supplementary Table 1**. At EBSI onset, 162 patients (84.4%) had neutropenia, of whom 145 had severe neutropenia. The median duration of neutropenia prior to EBSI onset was 12.0 days (IQR: 4.0–19.3). Most infections were nosocomial (n=184, 95.8%). The predominant infection type was MBI-LCBI (n=140, 72.9%), followed by secondary EBSI (n=34, 17.7%), most commonly of gastrointestinal origin (n=15). Ninety-three patients (48.4%) had pneumonia (of any etiology) at the time of EBSI onset, which was associated with significantly higher 30-day mortality compared to those without pneumonia (30.1% [28/93] *vs*. 15.2% [15/99], p=0.013). Among these patients, microbiologically confirmed pulmonary pathogens were identified in 12 cases, whereas the remaining cases were diagnosed based on clinical and radiological findings. The identified pathogens were heterogeneous, involving Gram-negative bacteria (including *Acinetobacter baumannii*, *Pseudomonas aeruginosa* and *Stenotrophomonas maltophilia*) and fungal pathogens (Candida species and molds). Regarding antimicrobial susceptibility, 67.7% of isolates (n=130) were resistant to ampicillin, including 126 *E. faecium* and 4 other *Enterococcus* species; all *E. faecalis* isolates were susceptible. Vancomycin resistance was rare, detected in only 3 isolates (1.6%), all *E. faecium*, indicating a low prevalence of vancomycin resistance in this cohort.

A total of 138 patients (71.9%) developed breakthrough EBSI while receiving antibiotics, with a median antibiotic exposure of 9.0 days (IQR, 5.0–12.0). The antibiotics administered at the time of breakthrough infection and their duration are summarized in **Supplementary Table 2**. Breakthrough infections occurred most frequently during carbapenem therapy (n=71, 51.4%), with a median duration of 9.0 days (IQR, 5.5–12.5), followed by cephalosporins (n=29, 21.0%) with a median duration of 9.0 days (IQR, 7.0–12.0). Metastatic infection developed in 9 patients (4.7%), involving soft tissues (n=5), lungs (n=3) and the abdominal cavity (n=1). Echocardiography was performed in 44 patients as clinically indicated, and no cases of metastatic endocarditis were identified. Persistent bacteremia was observed in 15 patients (7.8%), and septic shock in 12 patients (6.3%). Among patients with septic shock, 11 died within 30 days whereas only 1 survived, demonstrating a significant association with 30-day mortality (91.7% [11/12] *vs*. 17.8% [32/180], p<0.001).

### Treatment and clinical outcomes

The vast majority of patients (92.2%, n=177) received appropriate antibiotic therapy within 72 hours of blood culture collection. The antibiotics used for treatment are summarized in **Supplementary Table 3**, with linezolid and vancomycin being the most frequently administered agents for EBSI in our center. The median duration of antibiotics was 12.0 days (IQR: 8.0–17.0).

All-cause mortality rates at 14 and 30 days were 13.5% (n=26) and 22.4% (n=43), respectively. Infection-related mortality was 17.7% (n=34), and the 90-day recurrence rate was 3.1% (n=6). In univariate analysis, factors significantly associated with 30-day mortality included age ≥50 years (p=0.010), type of hematologic disease (p=0.042), severe GVHD (p=0.043), polymicrobial bacteremia (p=0.047), pneumonia at the time of EBSI (any pathogen) (p=0.013), septic shock (p<0.001), and duration of antibiotic therapy (p<0.001).

### Predictive model development and validation

LASSO logistic regression identified age ≥50 years, severe GVHD, septic shock, pneumonia, and type and stage of hematologic disease as candidate predictors with non-zero coefficients at the minimum cross-validated lambda. These variables were subsequently entered into a multivariable logistic regression model. To achieve a more parsimonious and clinically interpretable model, nested models were further evaluated. The final model included age ≥50 years, severe GVHD, septic shock, pneumonia, and high-risk hematologic disease, achieving an optimal balance between predictive accuracy, clinical interpretability, and model parsimony.

**Figure 2:**
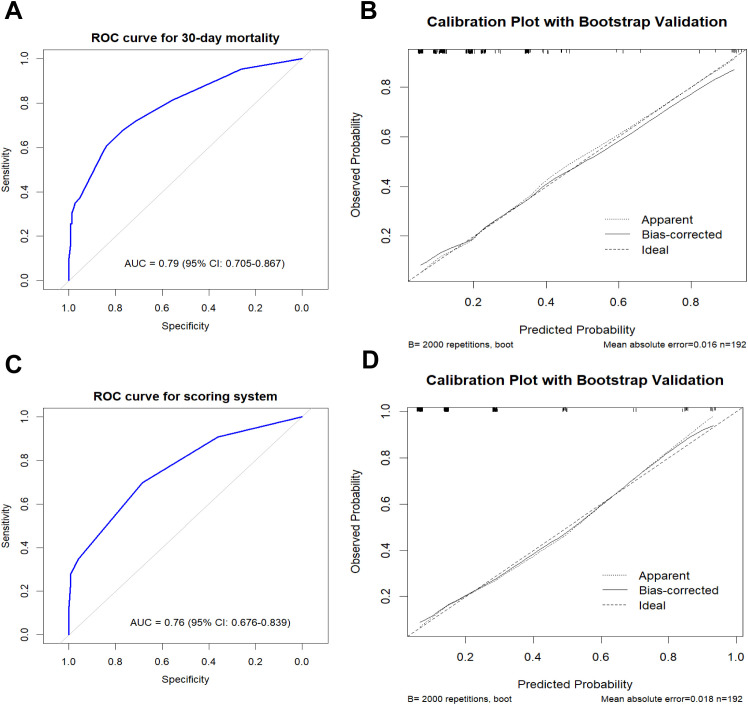
**(A)** ROC curve of the multivariable model for 30-day mortality. **(B)** Calibration plot of the multivariable model for 30-day mortality. **(C)** ROC curve of the scoring system; **(D)** Calibration plot of the scoring system.

In the final model, age ≥50 years (aOR=2.29, 95%CI 1.05-5.10, p=0.038), severe GVHD (aOR=6.06, 95%CI 1.86-19.96, p=0.003), septic shock (aOR=30.01, 95%CI 6.49-291.53, p<0.001) were independently associated with 30-day mortality. The model demonstrated good discrimination, with an AUROC of 0.79 (95% CI: 0.705–0.867) after 2000-bootstrap internal validation (**Figure 2A**). The Hosmer–Lemeshow goodness-of-fit test indicated excellent calibration (X²=0.994, df=5, P = 0.963), which was visually supported by the calibration plot (**Figure 2B**).

**Figure 3:**
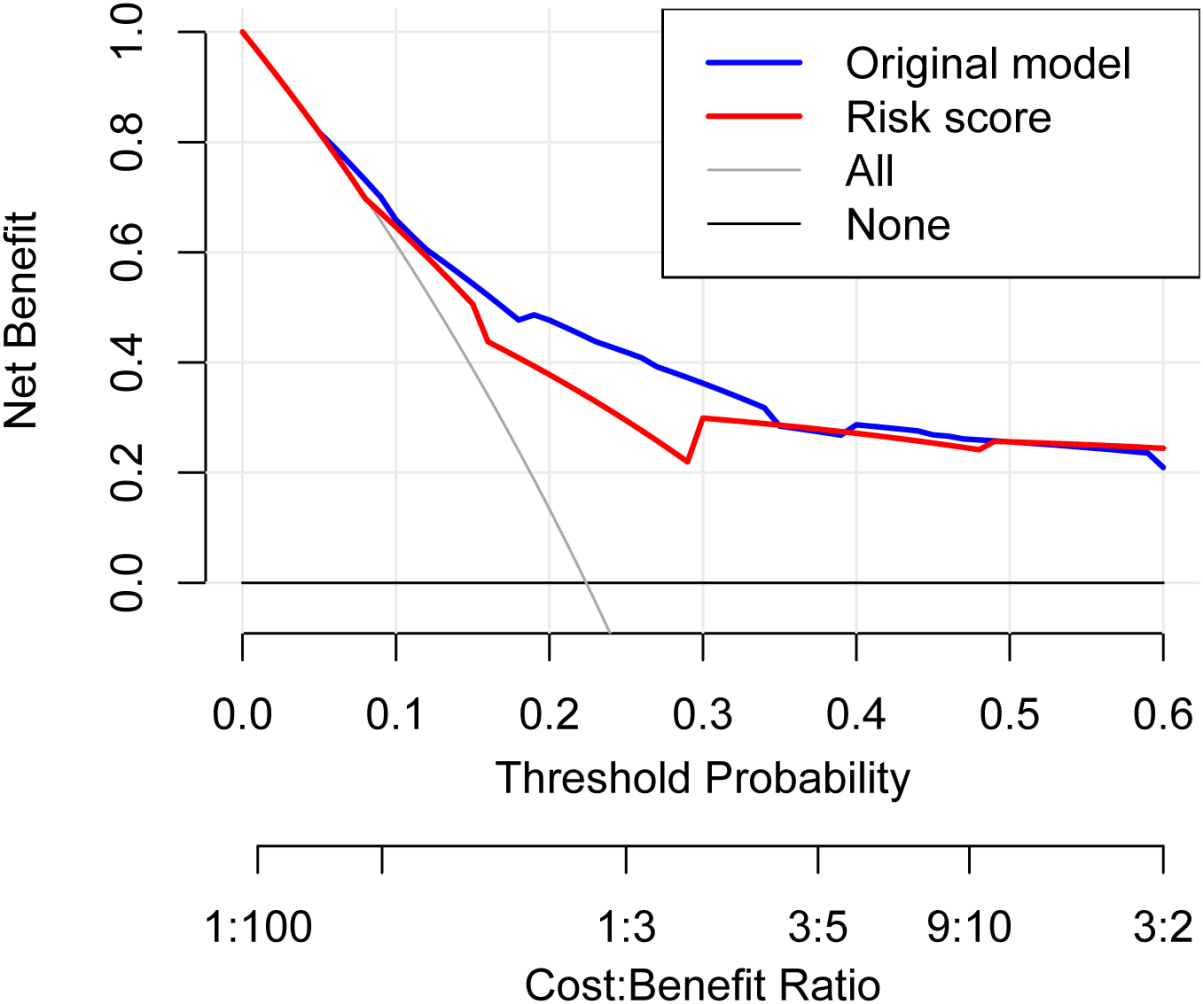
Decision curve analysis for the multivariable model and the scoring system.

**Figure 4:**
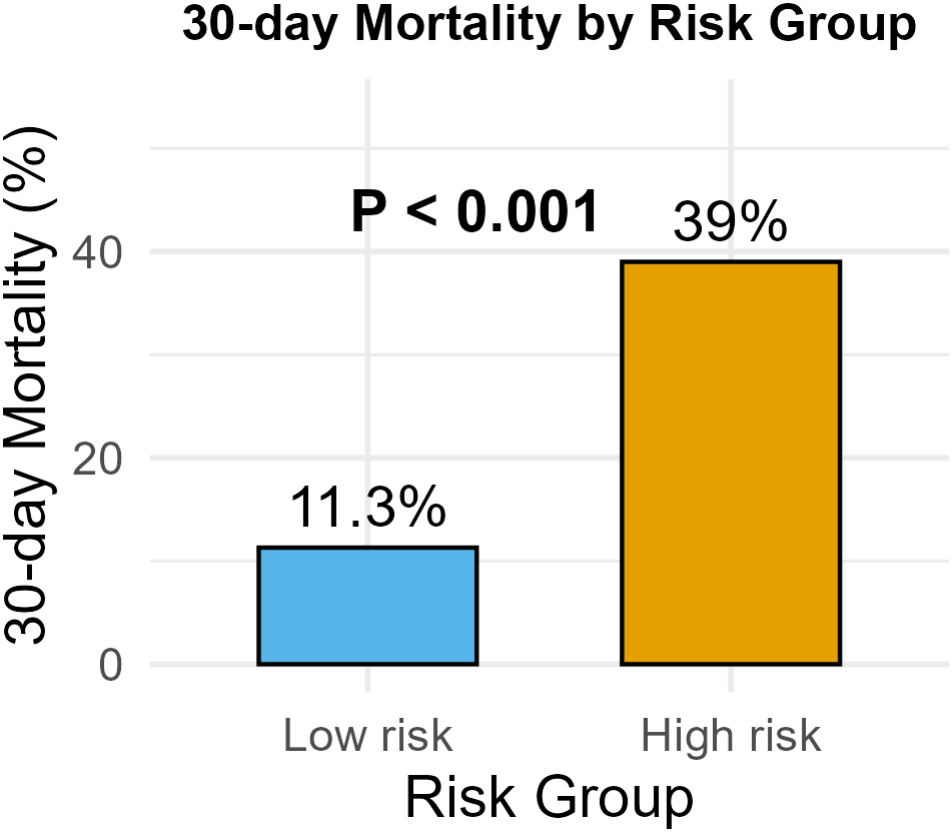
Risk stratification of hematologic patients with enterococcal bloodstream infection and corresponding 30-day mortality.

A simplified risk score was derived from regression coefficients: age ≥50 years (1 point), GVHD (2 points), septic shock (4 points), pneumonia (1 point) and high-risk hematologic disease (1 point) (**Table 2**). In bootstrap validation (B = 2000), the risk score achieved an AUROC of 0.76 (95% CI: 0.676-0.839), comparable to the original model (**Figure 2C**, 2D). DCA was conducted for both the multivariable logistic regression model and the simplified risk score. The DCA curves demonstrated potential clinical usefulness of the prediction tools (**Figure 3**). Using the optimal cutoff, patients were stratified into low-risk (<2 points) and high-risk (≥2 points) groups. The high-risk group had significantly higher 30-day mortality compared to the low-risk group (39.0% *vs*. 11.3%, P<0.001) (**Figure 4**).

**Table 2:** Risk scoring system for 30-day mortality in hematological patients with enterococcal bloodstream infection.

Variable	Coefficient	OR	Score
Age ≥50	0.83	2.29	1
Severe GVHD	1.80	6.06	2
Septic shock	3.40	30.01	4
Pneumonia	0.58	1.78	1
High risk hematologic disease	0.76	2.13	1

GVHD, graft-versus-host disease.

### Impact of antibiotic duration on 30-day mortality after minimizing immortal time bias

In univariable analysis (**Table 1**), duration of appropriate antibiotic therapy was significantly associated with 30-day mortality (p<0.001). To minimize immortal time bias, patients who died within 10 days of EBSI onset or received less than 5 days of appropriate antibiotic therapy were excluded, leaving 166 patients for further analysis. Multivariate logistic regression analysis showed that duration of appropriate antibiotic therapy was not significantly associated with mortality (OR = 0.95, 95%CI 0.88-1.00, p=0.076) (**Supplementary Table 4**).

## Discussion

EBSI is often associated with poor outcomes in patients with hematologic diseases and HSCT recipients. In this retrospective study, we found that EBSI predominantly occurred in neutropenic patients with mucosal barrier injury, frequently as a breakthrough infection during broad-spectrum antibiotic therapy, particularly carbapenems. We further identified age ≥50 years, severe GVHD and septic shock as independent predictors of 30-day mortality and developed a simple prognostic model that may facilitate early risk stratification and guide clinical decision-making.

Our findings confirm that EBSI remains a significant complication in hematologic patients, particularly those with acute leukemia or undergoing allo-HSCT. Over 70% of cases were caused by *E. faecium*, consistent with global trends highlighting its increasing significance in immunocompromised patients (Arias and Murray, 2012; Chen et al., 2017). Although ampicillin resistance was common, vancomycin resistance was unexpectedly low (1.6%), in contrast to many Western countries where VRE rates range from 4% to 35% (Vergis et al., 2001; Hornuss et al., 2024; Rinaldi et al., 2025; Zimmermann et al., 2025). However, it aligns with epidemiological data from China (0-3% (Zhang et al., 2017; Dai et al., 2022)) and certain European countries such as Denmark (Pinholt et al., 2014) and France (Souhail et al., 2019), where VRE prevalence remains similarly low (0-2%). Despite low resistance rate, the 30-day mortality in our cohort was notably high (22.4%) and significantly exceeded that of hematologic patients with *Staphylococcus aureus* bacteremia at our center during the same period (4.5%), where vancomycin resistance was also uncommon (Yang et al., 2025). These findings support previous observations that the poor prognosis of EBSI in immunocompromised patients is driven more by host vulnerability than by vancomycin resistance itself (Avery et al., 2005; Dubberke et al., 2006). Nevertheless, *E. faecalis* is known to harbor a wider array of virulence determinants (e.g., cytolysin, gelatinase, aggregation substance) (Ali et al., 2017; Archambaud et al., 2024), and the absence of species-level outcome differences in our study does not preclude a potential contribution of strain-specific virulence factors to infection severity. Future studies incorporating genomic characterization may help clarify whether bacterial pathogenic traits independently influence clinical outcomes in immunocompromised patients with EBSI.

In our study, nearly three-quarters of infections were classified as MBI-LCBIs, likely resulting from bacterial translocation across compromised mucosal barriers due to chemotherapy, HSCT or gastrointestinal GVHD (Dandoy et al., 2020; Liu et al., 2025). This highlights the importance of preserving mucosal integrity and mitigating gastrointestinal injury in high-risk patients undergoing chemotherapy or HSCT (Bowen et al., 2019; Elad et al., 2020). Notably, 71.9% of EBSI episodes occurred as breakthrough infections during antibiotic therapy, most frequently under carbapenems (51.4%), followed by cephalosporins (21.0%). This pattern likely results from antibiotic-induced disruption of the gut microbiota, which facilitates enterococcal overgrowth and translocation. The predominance of breakthrough EBSI during carbapenem therapy is consistent with previous studies demonstrating selective pressure favoring enterococcal colonization and infection (Zimmermann and Curtis, 2019; Webb et al., 2020). Clinicians should be alert to the possibility of EBSI in high-risk patients who develop fever or other signs of infection during carbapenem or cephalosporins therapy.

Consistent with previous studies, host-related factors such as advanced age and severe GVHD were significantly associated with increased mortality in immunocompromised patients with EBSI (Lisboa et al., 2015; Papanicolaou et al., 2019). In our analysis, severe GVHD was recorded only for allo-HSCT recipients. Therefore, severe GVHD could be interpreted as a conditional risk factor, reflecting a state of profound immune dysregulation, extensive mucosal barrier injury, and intensive immunosuppressive exposure. Septic shock, identified as the strongest predictor of death in our study, reflects profound systemic infection and immune dysfunction in this population and underscores the need for early hemodynamic stabilization and intensive care (Bauer et al., 2020; Lupia et al., 2022). Although pneumonia and high-risk hematologic disease were not statistically significant in multivariate logistic analysis, their inclusion improved model discrimination and calibration, suggesting that these features may still contribute important prognostic information. Similarly, Todeschini et al. analyzed 98 neutropenic patients with hematologic malignancies who developed EBSI and identified pneumonia of any etiology as the only independent risk factor for EBSI-related mortality (OR = 7.2, 95% CI 2.52–20.88, p=0.002) (Todeschini et al., 2006). This observation aligns with the fact that pulmonary involvement is a frequent and severe complication in hematologic patients (Guarana et al., 2019). In addition, consistent with our findings, both Bae et al. and Papanicolaou et al. demonstrated that advanced stages of hematologic malignancies were significantly associated with poorer overall survival in patients with EBSI (Bae et al., 2019; Papanicolaou et al., 2019).

To our knowledge, this is the largest cohort to develop a mortality prediction model specifically for hematologic patients with EBSI. The model incorporates five readily available clinical variables—age ≥50 years, severe GVHD, pneumonia, septic shock, and high-risk hematologic disease—enabling rapid risk assessment in routine practice. Internal validation confirmed good discrimination and calibration. Stratification into low- and high-risk groups can inform clinical decision-making: high-risk patients could be prioritized for intensive monitoring, early adjustment or escalation of antimicrobial therapy, and consideration of novel agents, whereas recognition of low-risk patients may help avoid overtreatment and enable more efficient use of healthcare resources.

Building upon the proposed risk stratification, we propose a preliminary therapeutic framework for high-risk patients with EBSI. High-risk patients (risk score ≥2 points), particularly those with septic shock or severe GVHD, may benefit from the following considerations: (1) In the setting of septic shock, empiric combination anti-enterococcal therapy (e.g., daptomycin plus a β-lactam) merits individualized consideration, informed by evidence demonstrating that combination therapy substantially reduces the pharmacodynamic threshold for daptomycin efficacy (Smith et al., 2015b; Smith et al., 2015a), as well as clinical data suggesting survival benefits of combination therapy in neutropenic patients with septic shock (Chumbita et al., 2022); (2) In cases without clinical improvement within 48–72 hours, vancomycin susceptibility should be reassessed, and early transition to daptomycin (≥10 mg/kg/day) (Britt et al., 2017) or linezolid (Hashemian et al., 2018) should be considered; in regions with prevalent vanB genotypes, teicoplanin may be a viable alternative (Xie et al., 2020); (3) Aggressive source control should be pursued, including early consideration of central venous catheter removal. Conversely, for low-risk patients (score <2 points), unnecessary combination therapy and prolonged antibiotic courses should be avoided. A 9-day short-course regimen is recommended (Bahrs et al., 2023), and catheter retention may be attempted in carefully selected patients. We emphasize that these strategies are primarily informed by indirect evidence and retrospective analyses, and warrant prospective validation.

Most existing prognostic models for bacteremia in hematologic patients focus primarily on Gram-negative infections. Although a few studies included Enterococcus, its proportion was very low (3%-7%) (Tang et al., 2018; Wang et al., 2023; Li et al., 2025). In contrast, our study specifically developed a scoring system tailored to EBSI in hematologic patients. This tool is simple, practical, and suitable for bedside application, with clearly defined and easily measurable predictors. The model demonstrated good performance in stratifying risk among patients with EBSI.

Moreover, our study also demonstrated that appropriate antimicrobial therapy given later than 72 hours after blood culture collection was not associated with higher mortality. Bussini et al. conducted a retrospective multicenter study of 758 hospitalized patients with EBSI, comparing outcomes between those who received active anti-enterococcal empirical therapy within 48 hours of the first blood culture (n=342) and those who did not (n=416) (Bussini et al., 2025). No significant mortality reduction was observed, either in the crude analysis (p=0.114) or after adjustment using inverse probability of treatment weighting (p=0.184) (Bussini et al., 2025). These findings are consistent with current febrile neutropenia guidelines, which do not recommend routine empirical coverage for Enterococcus in the absence of specific clinical indications (Freifeld et al., 2011).

Several limitations should be acknowledged. First, the single-center retrospective study design and lack of external validation limit the generalizability of the prediction model, despite its good performance in internal bootstrap validation. Second, the low VRE prevalence necessitates caution when applying these findings to settings with higher resistance rates. Third, we did not assess certain potentially relevant factors, such as prior enterococcal colonization, strain-specific virulence determinants or antibiotic therapeutic drug monitoring. Therefore, multicenter prospective studies are warranted to validate and refine the model. Despite these limitations, our study provides valuable preliminary evidence supporting future large-scale prospective investigations and offers insights that may inform the optimization of EBSI management in hematologic patients.

In conclusion, our study provides novel insights into the epidemiology, risk factors, and outcomes of EBSI in hematologic patients. *Enterococcus faecium* was the leading pathogen, with a low prevalence of vancomycin resistance. In those receiving chemotherapy, with gastrointestinal GVHD, or with persistent neutropenia, disruption of the mucosal barrier likely facilitates bacterial translocation, explaining why most EBSI episodes were classified as MBI-LCBIs and emphasizing the need to consider EBSI when fever or other signs of infection develop during carbapenem or cephalosporin therapy. A simple predictive model was developed based on five factors: older age, severe GVHD, pneumonia, septic shock and high-risk hematologic disease, which demonstrated good discriminative performance. Further multicenter prospective studies are warranted to externally validate and refine this scoring system. Ultimately, such efforts may help improve outcomes in this highly vulnerable patient population.

## Data Availability

The original contributions presented in the study are included in the article/**Supplementary Material**. Further inquiries can be directed to the corresponding authors.

## Supplementary material

Supplementary Table 1 Microbial composition of polymicrobial bacteraemia episodes in patients with enterococcal bloodstream infection.

Supplementary Table 2 Antibiotics administered at the time of breakthrough enterococcal bloodstream infection.

Supplementary Table 3 Summary of appropriate antibiotic therapy for enterococcal bloodstream infection.

Supplementary Table 4 Univariable and multivariable logistic regression analysis of 30-day mortality after excluding patients who died within 10 days of bacteremia onset or received less than 5 days of appropriate antibiotic therapy.

## References

[B1] Ali L. Goraya M. U. Arafat Y. Ajmal M. Chen J. L. Yu D. (2017). Molecular mechanism of quorum-sensing in enterococcus faecalis: its role in virulence and therapeutic approaches. Int. J. Mol. Sci. 18, 960. doi: 10.3390/ijms18050960, PMID: 28467378 PMC5454873

[B2] Archambaud C. Nunez N. da Silva R. A. G. Kline K. A. Serror P. (2024). Enterococcus faecalis: an overlooked cell invader. Microbiol. Mol. Biol. Rev. 88, e0006924. doi: 10.1128/mmbr.00069-24, PMID: 39239986 PMC11426025

[B3] Arias C. A. Murray B. E. (2012). The rise of the Enterococcus: beyond vancomycin resistance. Nat. Rev. Microbiol. 10, 266–278. doi: 10.1038/nrmicro2761, PMID: 22421879 PMC3621121

[B4] Avery R. Kalaycio M. Pohlman B. Sobecks R. Kuczkowski E. Andresen S. . (2005). Early vancomycin-resistant enterococcus (VRE) bacteremia after allogeneic bone marrow transplantation is associated with a rapidly deteriorating clinical course. Bone Marrow Transplant 35, 497–499. doi: 10.1038/sj.bmt.1704821, PMID: 15640812

[B5] Bae K. S. Shin J. A. Kim S. K. Han S. B. Lee J. W. Lee D. G. . (2019). Enterococcal bacteremia in febrile neutropenic children and adolescents with underlying Malignancies, and clinical impact of vancomycin resistance. Infection 47, 417–424. doi: 10.1007/s15010-018-1260-z, PMID: 30565009

[B6] Bahrs C. Rieg S. Hennigs A. Hitzenbichler F. Brehm T. T. Rose N. . (2023). Short-course versus long-course antibiotic treatment for uncomplicated vancomycin-resistant enterococcal bacteraemia: a retrospective multicentre cohort study. Clin. Microbiol. Infect. 29, 200–207. doi: 10.1016/j.cmi.2022.08.023, PMID: 36087919

[B7] Bauer M. Gerlach H. Vogelmann T. Preissing F. Stiefel J. Adam D. (2020). Mortality in sepsis and septic shock in Europe, North America and Australia between 2009 and 2019— results from a systematic review and meta-analysis. Crit. Care 24, 239. doi: 10.1186/s13054-020-02950-2, PMID: 32430052 PMC7236499

[B8] Bowen J. M. Gibson R. J. Coller J. K. Blijlevens N. Bossi P. Al-Dasooqi N. . (2019). Systematic review of agents for the management of cancer treatment-related gastrointestinal mucositis and clinical practice guidelines. Support Care Cancer 27, 4011–4022. doi: 10.1007/s00520-019-04892-0, PMID: 31286233

[B9] Britt N. S. Potter E. M. Patel N. Steed M. E. (2017). Comparative effectiveness and safety of standard-, medium-, and high-dose daptomycin strategies for the treatment of vancomycin-resistant enterococcal bacteremia among veterans affairs patients. Clin. Infect. Dis. 64, 605–613. doi: 10.1093/cid/ciw815, PMID: 28011602

[B10] Bussini L. Bavaro D. F. Brunetta E. Carella F. Accornero S. Rosselli Del Turco E. . (2025). What is the impact of anti-enterococcal empirical therapy on survival of patients with enterococcal bloodstream infections? Clin. Infect. Dis., ciaf323. doi: 10.1093/cid/ciaf323, PMID: 40577528

[B11] Cattaneo C. Rieg S. Schwarzer G. Muller M. C. Blumel B. Kern W. V. (2021). Enterococcus faecalis bloodstream infection: does infectious disease specialist consultation make a difference? Infection 49, 1289–1297. doi: 10.1007/s15010-021-01717-3, PMID: 34716548 PMC8613167

[B12] Centers for Disease Control and Prevention (2025). National healthcare safety network (NHSN) patient safety component manual. Available online at: https://www.cdc.gov/nhsn/pdfs/pscmanual/pcsmanual_current.pdf (Accessed May 10, 2025).

[B13] Chen C. Y. Tien F. M. Sheng W. H. Huang S. Y. Yao M. Tang J. L. . (2017). Clinical and microbiological characteristics of bloodstream infections among patients with haematological Malignancies with and without neutropenia at a medical centre in northern Taiwan, 2008-2013. Int. J. Antimicrob. Agents 49, 272–281. doi: 10.1016/j.ijantimicag.2016.11.009, PMID: 28109554

[B14] Chumbita M. Puerta-Alcalde P. Gudiol C. Garcia-Pouton N. Laporte-Amargos J. Ladino A. . (2022). Impact of empirical antibiotic regimens on mortality in neutropenic patients with bloodstream infection presenting with septic shock. Antimicrob. Agents Chemother. 66, e0174421. doi: 10.1128/AAC.01744-21, PMID: 34843387 PMC8846468

[B15] Dai Z. Chen L. Y. Cai M. J. Yao Y. H. Zhu J. H. Fang L. L. . (2022). Clinical characteristics and microbiology of nosocomial enterococcal bloodstream infections in a tertiary-level hospital: a retrospective study, 2007-2019. J. Hosp Infect. 122, 203–210. doi: 10.1016/j.jhin.2022.01.011, PMID: 35085678

[B16] Dandoy C. E. Kim S. Chen M. Ahn K. W. Ardura M. I. Brown V. . (2020). Incidence, risk factors, and outcomes of patients who develop mucosal barrier injury-laboratory confirmed bloodstream infections in the first 100 days after allogeneic hematopoietic stem cell transplant. JAMA Netw. Open 3, e1918668. doi: 10.1001/jamanetworkopen.2019.18668, PMID: 31913492 PMC6991246

[B17] Dubberke E. R. Hollands J. M. Georgantopoulos P. Augustin K. DiPersio J. F. Mundy L. M. . (2006). Vancomycin-resistant enterococcal bloodstream infections on a hematopoietic stem cell transplant unit: are the sick getting sicker? Bone Marrow Transplant. 38, 813–819. doi: 10.1038/sj.bmt.1705530, PMID: 17057724

[B18] Elad S. Cheng K. K. F. Lalla R. V. Yarom N. Hong C. Logan R. M. . (2020). MASCC/ISOO clinical practice guidelines for the management of mucositis secondary to cancer therapy. Cancer 126, 4423–4431. doi: 10.1002/cncr.33100, PMID: 32786044 PMC7540329

[B19] Freifeld A. G. Bow E. J. Sepkowitz K. A. Boeckh M. J. Ito J. I. Mullen C. A. . (2011). Clinical practice guideline for the use of antimicrobial agents in neutropenic patients with cancer: 2010 update by the infectious diseases society of america. Clin. Infect. Diseases 52, e56–e93. doi: 10.1093/cid/cir073, PMID: 21258094

[B20] Guarana M. Nucci M. Nouér S. A. (2019). Shock and early death in hematologic patients with febrile neutropenia. Antimicrobial Agents Chemotherapy 63, e01250-19. doi: 10.1128/AAC.01250-19, PMID: 31405857 PMC6811434

[B21] Hashemian S. M. R. Farhadi T. Ganjparvar M. (2018). Linezolid: a review of its properties, function, and use in critical care. Drug Des. Devel Ther. 12, 1759–1767. doi: 10.2147/DDDT.S164515, PMID: 29950810 PMC6014438

[B22] Hornuss D. Gopel S. Walker S. V. Tobys D. Hacker G. Seifert H. . (2024). Epidemiological trends and susceptibility patterns of bloodstream infections caused by Enterococcus spp. in six German university hospitals: a prospectively evaluated multicentre cohort study from 2016 to 2020 of the R-Net study group. Infection 52, 1995–2004. doi: 10.1007/s15010-024-02249-2, PMID: 38684586 PMC11499396

[B23] Jagasia M. H. Greinix H. T. Arora M. Williams K. M. Wolff D. Cowen E. W. . (2015). National institutes of health consensus development project on criteria for clinical trials in chronic graft-versus-host disease: I. The 2014 diagnosis and staging working group report. Biol. Blood Marrow Transplant 21, 389–401.e1. doi: 10.1016/j.bbmt.2014.12.001, PMID: 25529383 PMC4329079

[B24] Li J. S. Sexton D. J. Mick N. Nettles R. Fowler V. G. Jr. Ryan T. . (2000). Proposed modifications to the Duke criteria for the diagnosis of infective endocarditis. Clin. Infect. Dis. 30, 633–638. doi: 10.1086/313753, PMID: 10770721

[B25] Li Q. Lin N. Wang Z. Chen Y. Xie Y. Wang X. . (2025). Machine learning-based prognostic model for bloodstream infections in hematological Malignancies using Th1/Th2 cytokines. BMC Infect. Dis. 25, 415. doi: 10.1186/s12879-025-10808-7, PMID: 40140749 PMC11948653

[B26] Lisboa L. F. Miranda B. G. Vieira M. B. Dulley F. L. Fonseca G. G. Guimaraes T. . (2015). Empiric use of linezolid in febrile hematology and hematopoietic stem cell transplantation patients colonized with vancomycin-resistant Enterococcus spp. Int. J. Infect. Dis. 33, 171–176. doi: 10.1016/j.ijid.2015.02.001, PMID: 25660090

[B27] Liu M. Jiang X. Pi Y. Chen M. Ren X. Dai X. . (2025). Mucosal barrier injury as an independent risk factor for laboratory-confirmed bloodstream infection in patients with hematological Malignancies: a real-world study. Eur. J. Med. Res. 30, 649. doi: 10.1186/s40001-025-02913-9, PMID: 40685351 PMC12278662

[B28] Lopera C. Monzó P. Aiello T. F. Chumbita M. Peyrony O. Gallardo-Pizarro A. . (2024). Prevalence and impact of multidrug-resistant bacteria in solid cancer patients with bloodstream infection: a 25-year trend analysis. Microbiol. Spectr. 12, e0296123. doi: 10.1128/spectrum.02961-23, PMID: 39194256 PMC11448387

[B29] Lopez-Cortes L. E. Del Toro M. D. Galvez-Acebal J. Bereciartua-Bastarrica E. Farinas M. C. Sanz-Franco M. . (2013). Impact of an evidence-based bundle intervention in the quality-of-care management and outcome of Staphylococcus aureus bacteremia. Clin. Infect. Dis. 57, 1225–1233. doi: 10.1093/cid/cit499, PMID: 23929889

[B30] Lupia T. Roberto G. Scaglione L. Shbaklo N. De Benedetto I. Scabini S. . (2022). Clinical and microbiological characteristics of bloodstream infections caused by Enterococcus spp. within internal medicine wards: a two-year single-centre experience. Intern. Emerg. Med. 17, 1129–1137. doi: 10.1007/s11739-022-02926-w, PMID: 35092582 PMC8799962

[B31] Misch E. A. Andes D. R. (2019). Bacterial infections in the stem cell transplant recipient and hematologic Malignancy patient. Infect. Dis. Clin. North Am. 33, 399–445. doi: 10.1016/j.idc.2019.02.011, PMID: 31005135

[B32] Papanicolaou G. A. Ustun C. Young J. H. Chen M. Kim S. Woo Ahn K. . (2019). Bloodstream infection due to vancomycin-resistant enterococcus is associated with increased mortality after hematopoietic cell transplantation for acute leukemia and myelodysplastic syndrome: A multicenter, retrospective cohort study. Clin. Infect. Dis. 69, 1771–1779. doi: 10.1093/cid/ciz031, PMID: 30649224 PMC6821199

[B33] Peterson W. J. Maya I. D. Carlton D. Estrada E. Allon M. (2009). Treatment of dialysis catheter-related Enterococcus bacteremia with an antibiotic lock: a quality improvement report. Am. J. Kidney Dis. 53, 107–111. doi: 10.1053/j.ajkd.2008.06.033, PMID: 18848379 PMC2614136

[B34] Pinholt M. Ostergaard C. Arpi M. Bruun N. E. Schonheyder H. C. Gradel K. O. . (2014). Incidence, clinical characteristics and 30-day mortality of enterococcal bacteraemia in Denmark 2006-2009: a population-based cohort study. Clin. Microbiol. Infect. 20, 145–151. doi: 10.1111/1469-0691.12236, PMID: 23647880

[B35] Rangaraj G. Granwehr B. P. Jiang Y. Hachem R. Raad I. (2010). Perils of quinolone exposure in cancer patients: breakthrough bacteremia with multidrug-resistant organisms. Cancer 116, 967–973. doi: 10.1002/cncr.24812, PMID: 20052728

[B36] Rhodes A. Evans L. E. Alhazzani W. Levy M. M. Antonelli M. Ferrer R. . (2017). Surviving sepsis campaign: international guidelines for management of sepsis and septic shock: 2016. Intensive Care Med. 43, 304–377. doi: 10.1007/s00134-017-4683-6, PMID: 28101605

[B37] Rinaldi M. Rancan I. Malerba F. Gatti M. Ancillotti L. Tazza B. . (2025). Enterococcus faecium bacteraemia: a multicentre observational study focused on risk factors for clinical and microbiological outcomes. J. Antimicrob. Chemother. 80, 2247–2256. doi: 10.1093/jac/dkaf197, PMID: 40577612 PMC12313462

[B38] Rosselli Del Turco E. Bartoletti M. Dahl A. Cervera C. Pericas J. M. (2021). How do I manage a patient with enterococcal bacteraemia? Clin. Microbiol. Infect. 27, 364–371. doi: 10.1016/j.cmi.2020.10.029, PMID: 33152537

[B39] Satlin M. J. Soave R. Racanelli A. C. Shore T. B. van Besien K. Jenkins S. G. . (2014). The emergence of vancomycin-resistant enterococcal bacteremia in hematopoietic stem cell transplant recipients. Leuk Lymphoma 55, 2858–2865. doi: 10.3109/10428194.2014.896007, PMID: 24559288 PMC4316723

[B40] Schoemans H. M. Lee S. J. Ferrara J. L. Wolff D. Levine J. E. Schultz K. R. . (2018). EBMT–NIH–CIBMTR Task Force position statement on standardized terminology & guidance for graft-versus-host disease assessment. Bone Marrow Transplant 53, 1401–1415. doi: 10.1038/s41409-018-0204-7, PMID: 29872128 PMC6786777

[B41] Smith J. R. Barber K. E. Raut A. Aboutaleb M. Sakoulas G. Rybak M. J. (2015a). beta-Lactam combinations with daptomycin provide synergy against vancomycin-resistant Enterococcus faecalis and Enterococcus faecium. J. Antimicrob. Chemother. 70, 1738–1743. doi: 10.1093/jac/dkv007, PMID: 25645208 PMC4542582

[B42] Smith J. R. Barber K. E. Raut A. Rybak M. J. (2015b). beta-Lactams enhance daptomycin activity against vancomycin-resistant Enterococcus faecalis and Enterococcus faecium in *in vitro* pharmacokinetic/pharmacodynamic models. Antimicrob. Agents Chemother. 59, 2842–2848. doi: 10.1128/AAC.00053-15, PMID: 25753639 PMC4394769

[B43] Souhail B. Le Marechal M. Manuello R. Chretien R. Charlot P. Deroudilhes G. . (2019). Antibiotic therapy for Enterococcus bacteraemia: warning for the antimicrobial stewardship team. Eur. J. Clin. Microbiol. Infect. Dis. 38, 2087–2095. doi: 10.1007/s10096-019-03645-5, PMID: 31350634

[B44] Tang Y. Cheng Q. Yang Q. Liu J. Zhang D. Cao W. . (2018). Prognostic factors and scoring model of hematological Malignancies patients with bloodstream infections. Infection 46, 513–521. doi: 10.1007/s15010-018-1151-3, PMID: 29767394

[B45] Todeschini G. Tecchio C. Borghero C. D’Emilio A. Pegoraro E. de Lalla F. . (2006). Association between Enterococcus bacteraemia and death in neutropenic patients with haematological Malignancies. J. Infect. 53, 266–273. doi: 10.1016/j.jinf.2005.11.012, PMID: 16388852

[B46] Vergis E. N. Hayden M. K. Chow J. W. Snydman D. R. Zervos M. J. Linden P. K. . (2001). Determinants of vancomycin resistance and mortality rates in enterococcal bacteremia. a prospective multicenter study. Ann. Intern. Med. 135, 484–492. doi: 10.7326/0003-4819-135-7-200110020-00007, PMID: 11578151

[B47] Vydra J. Shanley R. M. George I. Ustun C. Smith A. R. Weisdorf D. J. . (2012). Enterococcal bacteremia is associated with increased risk of mortality in recipients of allogeneic hematopoietic stem cell transplantation. Clin. Infect. Dis. 55, 764–770. doi: 10.1093/cid/cis550, PMID: 22693346 PMC3657510

[B48] Wang L. Wang Y. Fan X. Tang W. Hu J. (2015). Prevalence of resistant gram-negative bacilli in bloodstream infection in febrile neutropenia patients undergoing hematopoietic stem cell transplantation. Medicine 94, e1931. doi: 10.1097/MD.0000000000001931, PMID: 26559260 PMC4912254

[B49] Wang J. Wang M. Zhao A. Zhou H. Mu M. Liu X. . (2023). Microbiology and prognostic prediction model of bloodstream infection in patients with hematological Malignancies. Front. Cell Infect. Microbiol. 13, 1167638. doi: 10.3389/fcimb.2023.1167638, PMID: 37457950 PMC10347389

[B50] Webb B. J. Majers J. Healy R. Jones P. B. Butler A. M. Snow G. . (2020). Antimicrobial stewardship in a hematological Malignancy unit: carbapenem reduction and decreased vancomycin-resistant enterococcus infection. Clin. Infect. Dis. 71, 960–967. doi: 10.1093/cid/ciz900, PMID: 31751470

[B51] Xie O. Slavin M. A. Teh B. W. Bajel A. Douglas A. P. Worth L. J. (2020). Epidemiology, treatment and outcomes of bloodstream infection due to vancomycin-resistant enterococci in cancer patients in a vanB endemic setting. BMC Infect. Dis. 20, 228. doi: 10.1186/s12879-020-04952-5, PMID: 32188401 PMC7079500

[B52] Yang N. Wang H. Feng X. Lin Q. Chen B. Mi Y. . (2025). Clinical characteristics and efficacy of short-course antibiotic therapy for Staphylococcus aureus bacteremia in hematological patients. Microbiol. Spectr. 13, e0232524. doi: 10.1128/spectrum.02325-24, PMID: 40793759 PMC12403743

[B53] Zhang Y. Du M. Chang Y. Chen L. A. Zhang Q. (2017). Incidence, clinical characteristics, and outcomes of nosocomial Enterococcus spp. bloodstream infections in a tertiary-care hospital in Beijing, China: a four-year retrospective study. Antimicrob. Resist. Infect. Control 6, 73. doi: 10.1186/s13756-017-0231-y, PMID: 28680588 PMC5496248

[B54] Zhang L. Zhen S. Shen Y. Zhang T. Wang J. Li J. . (2023). Bloodstream infections due to Carbapenem-Resistant Enterobacteriaceae in hematological patients: assessment of risk factors for mortality and treatment options. Ann. Clin. Microbiol. Antimicrobials 22, 41. doi: 10.1186/s12941-023-00586-y, PMID: 37202758 PMC10197250

[B55] Zimmermann P. Curtis N. (2019). The effect of antibiotics on the composition of the intestinal microbiota - a systematic review. J. Infect. 79, 471–489. doi: 10.1016/j.jinf.2019.10.008, PMID: 31629863

[B56] Zimmermann V. Fourre N. Senn L. Guery B. Papadimitriou-Olivgeris M. (2025). Predictors of mortality of enterococcal bacteraemia and the role of source control interventions; a retrospective cohort study. Infection. 53, 2149–2158. doi: 10.1007/s15010-025-02561-5, PMID: 40402401 PMC12460546

