# Supplementary material for: Who is at risk? Clinical features and a predictive model for 30-day mortality in hematologic patients with enterococcal bloodstream infection
Source: Front Cell Infect Microbiol. 2026 Mar 3;16:1762404. doi: 10.3389/fcimb.2026.1762404 (PMC12992278; doi:10.3389/fcimb.2026.1762404)
Supplement: Supplementary Table 1 — Microbial composition of polymicrobial bacteraemia episodes in patients with enterococcal bloodstream infection. [file Table1.docx]

Who Is at Risk? Clinical Features and a Predictive Model for 30-Day Mortality in Hematologic Patients with Enterococcal Bloodstream Infection

Nuobing Yang^1,2^, Sisi Zhen^1,2^, Tingting Zhang^1,2^, Yuping Fan^1,2^, Qingsong Lin^1,2^, Yingchang Mi^1,2^, Yizhou Zheng^1,2^, Lugui Qiu^1,2^, Fengkui Zhang^1,2^, Erlie Jiang^1,2^, Mingzhe Han^1,2^, Zhijian Xiao^1,2^, Jianxiang Wang^1,2^, Sizhou Feng^1,2^**^†^**, Xin Chen^1,2^**^†^**

^1^State Key Laboratory of Experimental Hematology, National Clinical Research Center

for Blood Diseases, Haihe Laboratory of Cell Ecosystem, Institute of Hematology &

Blood Diseases Hospital, Chinese Academy of Medical Sciences & Peking Union Medical

College, Tianjin, China

^2^Tianjin Institutes of Health Science, Tianjin, China

**^†^** Sizhou Feng and Xin Chen are co-corresponding author.

Sizhou Feng: szfeng@ihcams.ac.cn

Xin Chen: [chenxin@ihcams.ac.cn](mailto:chenxin@ihcams.ac.cn)

Address: State Key Laboratory of Experimental Hematology, National Clinical Research Center for Blood Diseases, Haihe Laboratory of Cell Ecosystem, Institute of Hematology & Blood Diseases Hospital, Chinese Academy of Medical Sciences & Peking Union Medical College, Tianjin 300020, China

Supplementary Methods

A clinical risk score was derived from the regression coefficients of the final multivariable logistic model. The calculation proceeded as follows:

**Step 1: Select a reference coefficient**

The coefficient of age ≥50 ($\beta_{age}$) was chosen as the reference because it is a core clinical predictor and provides a clinically interpretable anchor for scaling.

**Step 2: Compute relative scores for each predictor**

Each predictor’s regression coefficient ($\beta_{i}$) was divided by the reference coefficient to obtain a scaled value:

$${scaled score}_{i}= \frac{\beta_{i}}{\beta_{age}}$$

**Step 3: Round to integer values**

The scaled score for each predictor was rounded to the nearest integer to create a point-based score:

$${point}_{i=}round ({scaled score}_{i})$$

**Step 4: Convert predictors to numeric 0/1 variables**

For each patient, binary predictors were coded as 0 (absent) or 1 (present).

**Step 5: Calculate total patient score**

The total score for each patient was the sum of all predictor points multiplied by their presence indicators:

$$total score = \sum_{i} {point}_{i} \times X_{i}$$

Where $X_{i}=1$if predictor i is present, and 0 if absent.

**Step 6: Risk stratification**

Patients were stratified into low- and high-risk groups according to the total score, using cutoffs determined by receiver operating characteristic (ROC) curve analysis.

Supplemental Table 1 Microbial composition of polymicrobial bacteraemia episodes in patients with enterococcal bloodstream infection

| Pathogen | Number of cases, n |
| --- | --- |
| *Escherichia coli* | 9 |
| *Klebsiella pneumoniae* | 8 |
| *Pseudomonas aeruginosa* | 4 |
| *Staphylococcus haemolyticus* | 3 |
| *Acinetobacter baumannii* | 2 |
| *Staphylococcus aureus* | 2 |
| *Elizabethkingia meningoseptica* | 2 |
| *Proteus mirabilis* | 1 |
| *Staphylococcus epidermidis* | 1 |
| *Stenotrophomonas maltophilia* | 1 |
| *Trichosporon asahii* | 1 |

Supplemental Table 2 Antibiotics administered at the time of breakthrough enterococcal bloodstream infection

| Antibiotics break | Total  (n=138) | *E. faecalis*  (n=23) | *E. faecium*  (n=108) | Other  (n=7) | Median, IQR |
| --- | --- | --- | --- | --- | --- |
| Carbapenems | 71 | 6 | 62 | 3 | 9.0 (5.5, 12.5) |
| Cephalosporins | 29 | 13 | 13 | 3 | 9.0 (7.0, 12.0) |
| BLBLIs | 24 | 4 | 19 | 1 | 5.5 (4.0, 11.2) |
| Fluoroquinolones | 4 | 0 | 4 | 0 | 11 (9.3, 11.5) |
| Contezolid | 4 | 0 | 4 | 0 | 9 (6.5, 11) |
| Glycopeptides | 3 | 0 | 3 | 0 | 8 (6.5, 16.5) |
| Tigecycline | 2 | 0 | 2 | 0 | 9.5 (7.8, 11.2) |
| Daptomycin | 1 | 0 | 1 | 0 | 3.0 (3.0, 3.0) |

BLBLIs, β-lactam-β-lactamase inhibitor combinations

Supplemental Table 3 Summary of appropriate antibiotic therapy for enterococcal bloodstream infection

| Antibiotics | *E. faecalis*  (n=40) | *E. faecium*  (n=137) | Others  (n=15) |
| --- | --- | --- | --- |
| Linezolid | 8 | 41 | 3 |
| Vancomycin | 10 | 39 | 1 |
| Teicoplanin | 6 | 16 | 3 |
| Daptomycin | 5 | 19 | 1 |
| Contezolid | 4 | 15 | 1 |
| Tigecycline | 2 | 3 | 1 |
| Meropenem | 2 | 0 | 4 |
| Imipenem | 1 | 0 | 0 |
| Eravacycline | 0 | 3 | 0 |
| Cefoperazone-tazobactam | 0 | 0 | 1 |
| Moxifloxacin | 1 | 0 | 0 |
| Combination | 1 | 1 | 0 |

Combination: vancomycin + contezolid for *E. faecium*; daptomycin + linezolid for *E. faecalis*.

Supplementary Table 4 Univariable and multivariable logistic regression analysis of 30-day mortality after excluding patients who died within 10 days of bacteremia onset or received less than 5 days of appropriate antibiotic therapy

| Type of variable, characteristic | Univariate  OR (95%CI) | | P | Multivariate  OR (95%CI) | P |
| --- | --- | --- | --- | --- | --- |
| Age ≥50 | 2.28 (0.89, 6.00) | | 0.088 |  |  |
| Male | 1.53 (0.59, 4.28) | | 0.391 |  |  |
| CCI | 1.35 (0.76, 2.48) | | 0.315 |  |  |
| Type of hematologic disease |  | |  |  |  |
| Bone marrow failure syndromes | 1 | |  |  |  |
| Acute leukemia | 0.40 (0.14, 1.17) | | 0.083 |  |  |
| Other hematological diseases | 1.00 (0.13, 5.21) | | 1.000 |  |  |
| Stage of underlying diseases |  | |  |  |  |
| Standard risk | 1 | |  |  |  |
| High risk | 1.98 (0.77, 5.32) | | 0.161 |  |  |
| Allo-HSCT | 0.54 (0.12, 1.72) | | 0.346 |  |  |
| Severe GVHD | 5.75 (1.57, 19.63) | | 0.006 | 7.70 (1.86, 33.53) | 0.006 |
| Auto-HSCT | - | | 0.992 |  |  |
| Chemotherapy or immunosuppressive therapy within 1 month prior to BSI | 0.59 (0.14, 4.08) | | 0.522 |  |  |
| Microbiology |  | |  |  |  |
| Enterococcus faecalis | 1 | |  |  |  |
| Enterococcus faecium | 0.82 (0.29, 2.71) | | 0.729 |  |  |
| Other enterococci | 0.43 (0.02, 3.00) | | 0.458 |  |  |
| Ampicillin resistance | 0.91 (0.35, 2.56) | | 0.850 |  |  |
| Vancomycin resistance | 3.79 (0.17, 41.42) | | 0.286 |  |  |
| Polymicrobial bacteremia | 1.41 (0.38, 4.28) | | 0.571 |  |  |
| Breakthrough bacteremia | 0.91 (0.34, 2.72) | | 0.858 |  |  |
| Source of infection |  | |  |  |  |
| Non-MBI primary BSI | 1 | | 1 |  |  |
| MBI-LCBI | 0.98 (0.24, 6.64) | | 0.981 |  |  |
| Secondary BSI | 0.48 (0.05, 4.38) | | 0.489 |  |  |
| Nosocomial infection | 0.32 (0.06, 2.34) | | 0.191 |  |  |
| Metastatic infection | - | | 0.991 |  |  |
| Persistent bacteremia | 3.40 (0.86, 11.54) | | 0.059 |  |  |
| Septic shock | 13.11 (1.66, 149.09) | | 0.017 | 20.45 (1.86, 314.74) | 0.014 |
| Pneumonia | 3.51 (1.32, 10.69) | | 0.011 | 5.36 (1.83, 19.14) | 0.002 |
| Days of neutropenia before BSI | 1.01 (0.99, 1.03) | | 0.365 |  |  |
| Days of neutropenia after BSI | 0.99 (0.96, 1.02) | | 0.704 |  |  |
| Inappropriate therapy within 24 h | | 0.95 (0.37, 2.50) | 0.922 |  |  |
| Inappropriate therapy within 48 h | | 1.28 (0.47, 3.30) | 0.613 |  |  |
| Inappropriate therapy within 72 h | | 0.65 (0.03, 3.61) | 0.684 |  |  |
| Duration of antibiotic therapy | 0.98 (0.91, 1.03) | | 0.438 | 0.95 (0.88, 1.00) | 0.076 |

CCI, Charlson Comorbidity Index; allo-HSCT, allogeneic hematopoietic stem cell transplantation; GVHD, graft-versus-host disease; auto-HSCT, autologous hematopoietic stem cell transplantation; BSI, bloodstream infection; MBI-LCBI, mucosal barrier injury laboratory- confirmed bloodstream infection; ANC, absolute neutrophil count.
